# Comparison of ambulatory blood pressure monitoring and office blood pressure in primary health care of populations at a high risk of hypertension

**DOI:** 10.3389/fpubh.2022.985730

**Published:** 2023-01-11

**Authors:** Wei Gao, Yanwen Jin, Ting Bao, Yan Huang

**Affiliations:** ^1^Health Management Center, General Practice Center, West China Hospital, Sichuan University, Chengdu, China; ^2^Biliary Surgery, West China Hospital, Sichuan University, Chengdu, China

**Keywords:** ambulatory blood pressure monitoring (ABPM), office blood pressure, hypertension, primary health care, high-risk population

## Abstract

**Introduction:**

Current studies have found that the incidence of masked hypertension is high in Asian countries, but the use of ambulatory blood pressure monitoring (ABPM) in Asian countries is very limited, especially in primary health care. We compared the ABPM and office blood pressure (OBP) in primary health care of a high-risk population of hypertension.

**Methods:**

The study included participants with at least one risk factor for hypertension who received primary health care. Demographic data, present medical history, personal history, and family history were collected by questionnaire.

**Results:**

A total of 823 subjects were included in the study. There were 531 (64.5%) subjects with hypertension by ABPM and 316 patients (38.4%) by OBP. A paired chi-square test showed that the positive rate of ABPM in the diagnosis of hypertension was significantly higher than that of OBP (chi-square value 174.129, *P* < 0.0001). There were 24 (2.9%) patients with white coat hypertension, 239 (29.0%) with masked hypertension, 504 (52.9%) with a non-dipping pattern, 135 (16.9%) with nocturnal hypertension and 18 (2.2%) with high ambulatory BP variability. Concordance correlation coefficient showed there was a poor correlation between OBP and awake average BP. Scatter plot displayed there was a positive correlation between OBP and awake average BP, but the degree of fitting was not high. The Bland Altman plot showed that OBP and awake average BP were consistent.

**Conclusions:**

Although OBP and ABPM have some consistency, ABPM can screen for masked hypertension and nocturnal hypertension in primary care of populations at high risk of hypertension. Therefore, ABPM is necessary in the primary health care of populations at high risk of hypertension and can be used as a routine screening.

## 1. Background

Hypertension is an important risk factor leading to cardiovascular disease and stroke, and it is an important public health problem (1). Risk factors for hypertension include age, smoking, alcoholism, low physical activity, high salt diet, family history of hypertension and overweight or obese (2). At present, office blood pressure (OBP) is still an important means of hypertension screening and management and is especially suitable for primary health care because of its convenience and economy (3, 4). However, OBP has great limitations. OBP is easily affected by the operation method, and variability is great (3). Multiple OBP measurements can minimize the impact of the limitations, but white-coat hypertension and masked hypertension are difficult to detect by OBP (3). A study involving 14,143 people in 27 countries showed that the prevalence rates of white coat hypertension and occult hypertension were 23% and 10%, respectively (5). With the sufficient understanding of the limitations of OBP and the importance of ambulatory blood pressure monitoring (ABPM), an increasing number of scholars are paying attention to the role of ABPM in the diagnosis, prognosis and management of hypertension. ABPM can evaluate BP at multiple time points in daily life and automatically calculate average BP, including 24-h average BP, daytime average BP and nighttime average BP. It can also evaluate the circadian rhythm of BP, which contains abundant BP parameters and is more conducive to finding masked hypertension (4, 6). Current studies have found that the incidence of masked hypertension, morning hypertension and nocturnal hypertension in Asian countries is higher than that in Western countries (7–9). However, the use of ABPM in Asian countries, including China, is very limited, especially in primary health care (10). The purpose of this study was to compare OBP with ABPM in high hypertension-risk Asian individuals in primary care.

## 2. Methods

### 2.1. Subjects

The population at high risk of hypertension who received primary health care in West China Hospital Health Management Center from November 2020 to October 2021 was included.

#### 2.1.1. Inclusion criteria

At least one of the following risks of hypertension was met (2). (1) Age ≥ 40 years old; (2) Smoking; (3) Alcoholism; (4) Low physical activity (11); (5) High salt diet; (6) Family history of hypertension; (7) Overweight or obese.

#### 2.1.2. Exclusion criteria

Patients with hypertension, severe cardiovascular and cerebrovascular diseases, severe liver and kidney diseases, severe mental illness and malignant tumors.

This study was approved by the Ethics Committee of West China Hospital, and the subjects signed the informed consent form.

### 2.2. Data collection and indicator measurement

Basic information was collected by questionnaire. The questionnaire included demographic data, present medical history, personal history and family history. The present medical history consisted of hypertension, diabetes, hyperlipidemia, cardiovascular and cerebrovascular diseases, malignant tumors, liver and kidney diseases, and mental illness. Personal history included smoking history, drinking history, exercise, and salt intake. Family history included a family history of hypertension. Height, weight, OBP and ABPM were measured on the same date.

#### 2.2.1. Height and weight measurement method

The subjects only wore single clothes and took off their shoes and socks. Height and weight were measured by a calibrated electronic meter (Seca 287, Germany).

#### 2.2.2. OBP measurement method

The subjects rested for at least 5 min, and no smoking, tea or coffee was taken within 30 min. BP was measured twice by a calibrated electronic sphygmomanometer (Omron HBP-9021, Japan) by professional nurses, and the average value was taken (12).

#### 2.2.3. ABPM method

ABPM is the gold standard measure of BP. ABPM equipment (SPACELABS, 90217A, America) was used to monitor 24-h BP and outputted average BP, average pulse pressure, average daytime BP, average nighttime BP and circadian rhythm. The ABPM equipment was installed by professional nurses in the hospital. Then, subjects took the device home, and the nurse removed it the next day. The hour between waking up and going to bed was defined as daytime, and the hour between going to bed and waking up was defined as nighttime (13).

### 2.3. Diagnostic criteria

#### 2.3.1. Phenotype hypertension

(1) Hypertension

*OBP* ≥140/90 mm Hg or ambulatory BP was elevated (24-h average *BP* ≥130/80 mm Hg or daytime average *BP* ≥135/85 mm Hg or nighttime average *BP* ≥120/70 mm Hg) (2).

(2) White-coat hypertension

Office *BP* ≥140/90 mm Hg and ambulatory BP were normal (24-h average *BP* < 130/80 mm Hg and daytime average *BP* < 135/85 mm Hg) (2).

(3) Masked hypertension

Office *BP* < 140/90 mm Hg and ambulatory BP were elevated (24-h average *BP* ≥130/80 mm Hg or daytime average *BP* ≥135/85 mm Hg) (2).

(4) Non-dipping pattern

Diel rhythm was < 10% (2).

(5) Nocturnal hypertension

Nighttime ambulatory BP average ≥120/70 mm Hg, 24-h average *BP* < 130/80 mm Hg and daytime average *BP* < 135/85 mm Hg (2).

(6) High ambulatory BP variability

Diel rhythm was more than 20% (2).

#### 2.3.2. Overweight and obesity

Overweight: 24 kg/m^2^ ≤ body mass index (BMI) < 28 kg/m^2^ (14);

Obesity: *BMI* ≥ 28 kg/m ^2^ (14).

#### 2.3.3. High salt diet

Salt consumption was investigated in a questionnaire. Taste for salty food was divided into very light, light, average, salty, and very salty. The average level of salt intake was 6 g/d. The taste for salty or very salty food (salt intake >6 g/d) was defined as a high salt diet (15).

### 2.4. Statistical methods

IBM SPSS 26.0 and MedCalc 19.8 software were used for statistical analysis. The continuity variable was expressed as the mean value ($\bar{X}$) ± standard deviation (S). Qualitative data were described by absolute numbers and rates. The rates of the two groups were compared using the paired chi-square test. The two continuity variables were compared using paired *t* tests. Concordance correlation coefficient, scatter chart, Bland Altman plot, ROC curve and area under the curve were used to compare OBP and awake average BP. α = 0.05.

## 3. Results

We screened 1,286 cases and excluded 463 cases according to the inclusion and exclusion criteria. A total of 823 subjects were included in the study, including 485 (58.9%) males and 338 (41.1%) females. The average age was 52.67 ± 11.76 (30–81) years old. There were 678 (82.4%) subjects aged 40 or older. The subjects were all Chinese. There were 95 (11.5%) patients with diabetes and 82 (10.0%) patients with dyslipidemia. Two hundred ninety-three (35.6%) subjects had a history of smoking, 98 (11.9%) had a history of drinking, 338 (41.1%) had a family history of hypertension, 442 (53.7%) were overweight or obese, 651 (79.1%) had low physical activity, and 319 (38.8%) had a high-salt diet. The number of hypertension cases was 316 (38.4%) by OBP and 531 (64.5%) by ABPM, and the paired chi-square test showed that the difference was statistically significant (chi-square value 174.129, *P* < 0.0001). There were 24 (2.9%) patients with white coat hypertension, 239 (29.0%) with masked hypertension, 504 (52.9%) with a non-dipping pattern, 135 (16.9%) with nocturnal hypertension and 18 (2.2%) with high ambulatory BP variability. The details are shown in Table 1.

**Table 1:** Baseline characteristics and prevalence of hypertension phenotypes in the participants.

**Variables**		**Results**
Gender	Male	485 (58.9%)
	Female	338 (41.1%)
Age (year)		52.67 ± 11.76
Age (≥40 year)		678 (82.4%)
Race	Asian	823 (100%)
Diabetes		95 (11.5%)
Dyslipidemia		82 (10.0%)
Smoking		293 (35.6%)
Drinking		98 (11.9%)
Family history of hypertension		338 (41.1%)
Overweight or obese		442 (53.7%)
Low physical activity		651 (79.1%)
High salt diet		319 (38.8%)
BMI (kg/m^2^)		25.24 ± 3.49
Hypertension (OBP)		316 (38.4%)
Hypertension (APBM)		531 (64.5%)
White coat hypertension		24 (2.9%)
Masked hypertension		239 (29.0%)
Non-dipping pattern		504 (52.9%)
Nocturnal hypertension		135 (16.9%)
High ambulatory BP variability		18 (2.2%)

The BP results of the included subjects are shown in Table 2. OBP was 136.06 ± 16.25/81.61 ± 12.30 mm Hg. The 24-h average BP was 126.88 ± 15.42/80.48 ± 11.25 mm Hg, the awake average BP was 130.56 ± 14.69/84.48 ± 12.60 mm Hg, and the nighttime average BP was 119.59 ± 16.33/75.50 ± 11.44 mm Hg.

**Table 2:** BP results of the included subjects.

**Variables (mm Hg)**	**Mean±standard deviation**
Office systolic pressure	136.06 ± 16.25
Office diastolic pressure	81.61 ± 12.30
24 h average systolic pressure	126.88 ± 15.42
24 h average diastolic pressure	80.48 ± 11.25
Awake average systolic pressure	130.56 ± 14.69
Awake average diastolic pressure	84.48 ± 12.60
Nighttime average systolic pressure	119.59 ± 16.33
Nighttime average diastolic pressure	75.50 ± 11.44

**Figure 1:**
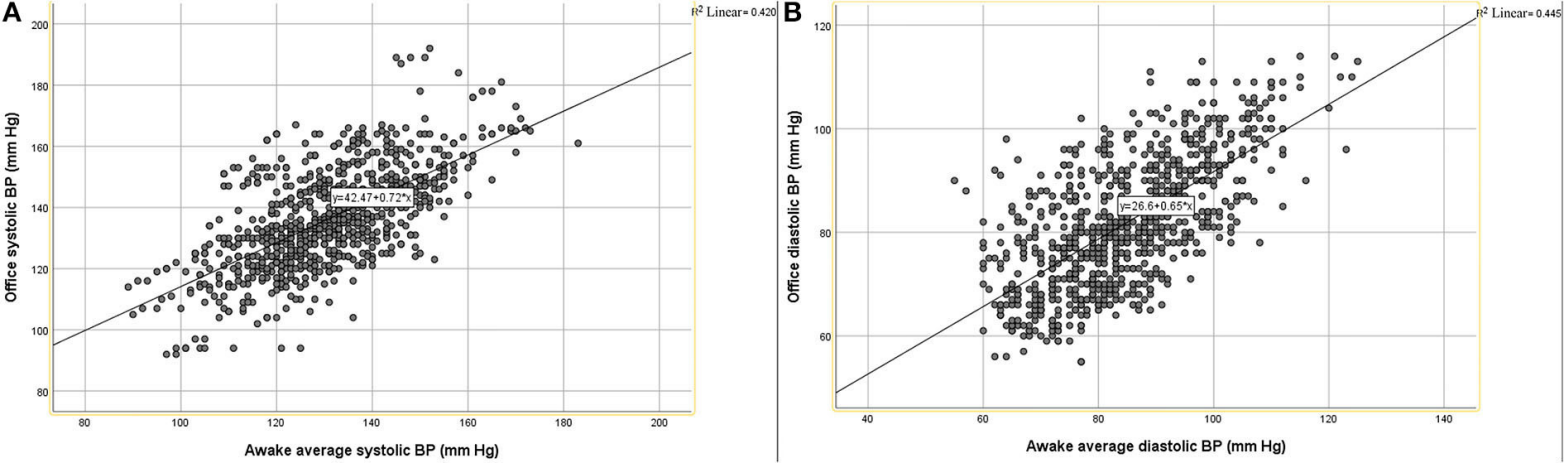
Scatter diagram of OBP and awake average BP. **(A)** Systolic pressure, **(B)** diastolic pressure.

**Figure 2:**
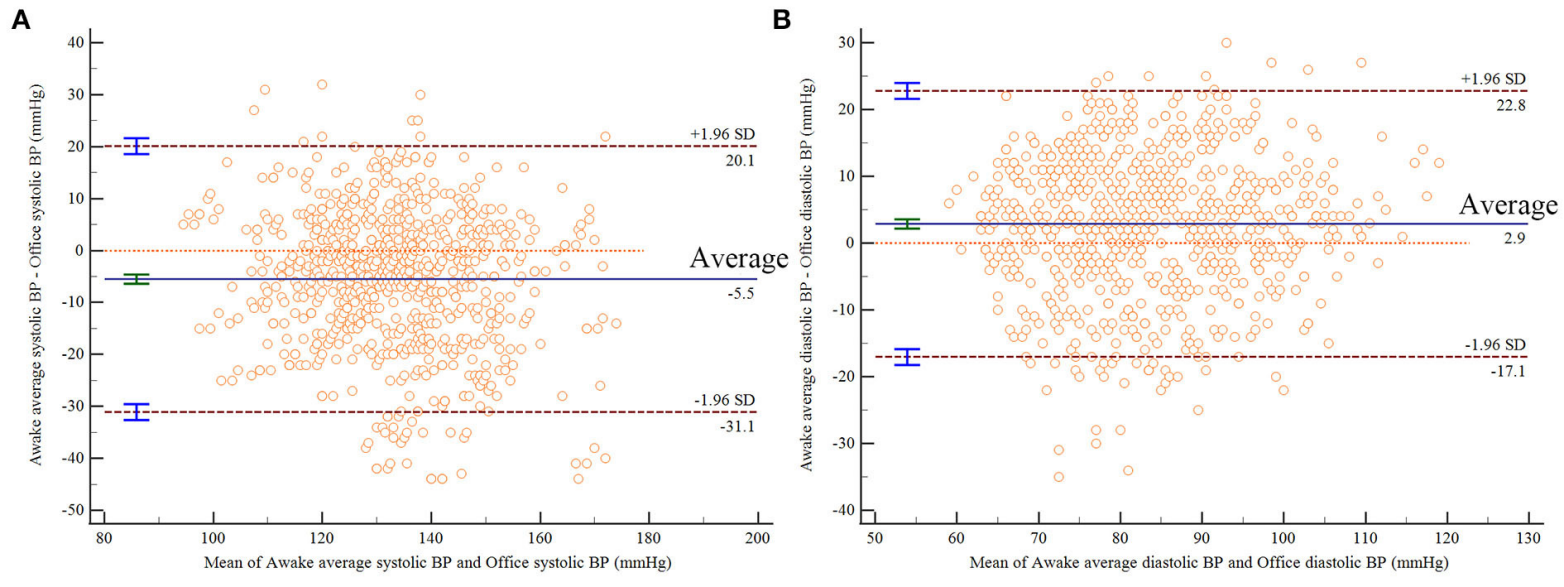
Bland Altman plot results of OBP and awake average BP. **(A)** Systolic pressure, **(B)** diastolic pressure.

**Figure 3:**
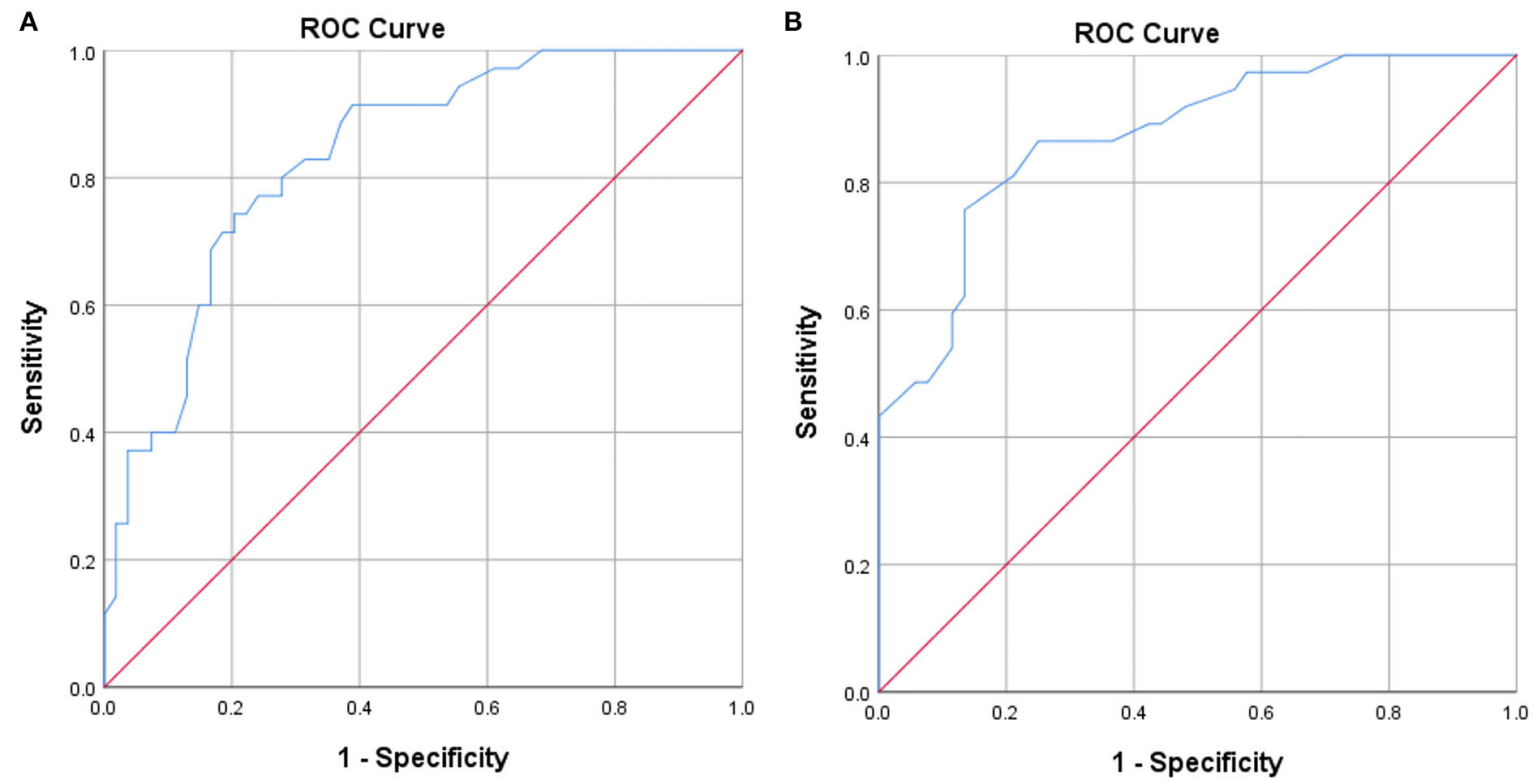
**(A, B)** ROC curves for OBP and awake average BP.

The correlation between office systolic pressure and awake average systolic pressure was poor (Concordance correlation coefficient = 0.606), and the same relationship was observed between office diastolic pressure and awake average diastolic pressure (Concordance correlation coefficient = 0.650). The scatter plot indicates that there was a positive correlation between office systolic pressure and awake average systolic pressure, but the degree of fitting was not high (R^2^ = 0.420, Figure 1A). There was a positive correlation between office diastolic pressure and awake average diastolic pressure, but the degree of fitting was also not high (R^2^ = 0.445, Figure 1B). The Bland Altman plot showed that office systolic pressure and awake average systolic pressure were consistent (*P* < 0.0001, Figure 2A), as were office diastolic pressure and awake average diastolic pressure (*P* < 0.0001, Figure 2B). The ROC curves of office systolic pressure and awake average systolic pressure is shown in Figure 3A, office diastolic pressure and awake average diastolic pressure in Figure 3B, and the areas under the ROC curves were 0.834 and 0.872, respectively.

## 4. Discussion

Previous studies on ABPM have mainly focused on outpatients or inpatients (16–18), and the number of studies on the population receiving primary health care is very limited. ABPM is not routinely used in primary health care due to its high cost and limited resources (10).

In this study, by comparing the values of OBP and ambulatory BP, it was found that there was a poor correlation between them, with consistency, and there was a linear relationship, but the degree of fit was not high. Although the OBP and ABPM have consistency, the positive rate of ABPM (64.5%) for screening hypertension was significantly higher than that of OBP (38.4%). In this study, the subjects were the high-risk population of hypertension, which may be the reason for the higher prevalence of hypertension than in the previous study (19).

ABPM screened out 24 (2.9%) patients with white coat hypertension, 239 (29.0%) with masked hypertension, 504 (52.9%) with a non-dipping pattern, 135 (16.9%) with nocturnal hypertension and 18 (2.2%) with high ambulatory BP variability. Previous studies have reported that the prevalence of white coat hypertension was 13% in untreated people (20, 21), the prevalence rate of masked hypertension in China is 17.8% (20), and the prevalence of nocturnal hypertension was approximately 10% in the Chinese population and 6.0% ~ 7.9% in Europe and America (22). This may be associated with salt-sensitive hypertension in the Chinese population (23). We hypothesize that the differences from previous studies are mainly due to race and the differences in the included population.

Although the cost increases at present, it will save the cost in the long run (24). The 24-h mean BP and nocturnal BP can better predict the morbidity and mortality of cardiovascular and cerebrovascular diseases (25). Patients with masked hypertension and nocturnal hypertension can receive intervention treatment as soon as possible, and some of them can be alleviated only by lifestyle intervention, which can greatly reduce the incidence and mortality of complications such as cardiovascular and cerebrovascular diseases, which can reduce the economic burden of patients' families and society in the long run (26–28). This study provides an important basis for the application of ABPM in the primary care of high-risk hypertension populations.

This study has some advantages. The population included in this study was at high risk for hypertension receiving primary care, and this population has been less studied in previous studies. This study is a prospective study design and is relatively more reliable than the results of cross-sectional studies. Of course, there are some drawbacks to this study. The study did not include a long follow-up period, and the sample size of this study was not large enough. Therefore, high-quality studies with a larger sample size and long-term follow-up are needed to further confirm the role and significance of ABPM in primary care.

## 5. Conclusion

Although OBP and ABPM haves some consistency, ABPM can screen for masked hypertension and nocturnal hypertension in primary care of populations at high risk of hypertension. Therefore, ABPM is necessary in the primary health care of populations at high risk of hypertension and can be used as a routine screening.

## Data availability statement

The original contributions presented in the study are included in the article/supplementary material, further inquiries can be directed to the corresponding authors.

## Ethics statement

The studies involving human participants were reviewed and approved by West China Hospital, Sichuan University. The patients/participants provided their written informed consent to participate in this study.

## Author contributions

WG was responsible for the study design, data collection, statistical analysis, and manuscript writing. YJ contributed to data collection. TB contributed to statistical analysis. YH was responsible for the study design and manuscript revision. All authors contributed to the article and approved the submitted version.
